# Supplementary material for: Involvement of Serotonergic Projections from the Dorsal Raphe to the Medial Preoptic Area in the Regulation of the Pup-Directed Paternal Response of Male Mandarin Voles
Source: Int J Mol Sci. 2023 Jul 18;24(14):11605. doi: 10.3390/ijms241411605 (PMC10380723; doi:10.3390/ijms241411605)
Supplement: Supplementary file 1 [file ijms-24-11605-s001.zip › ijms-2450485-supplementary.pdf]

Supplementary information

Figure S1

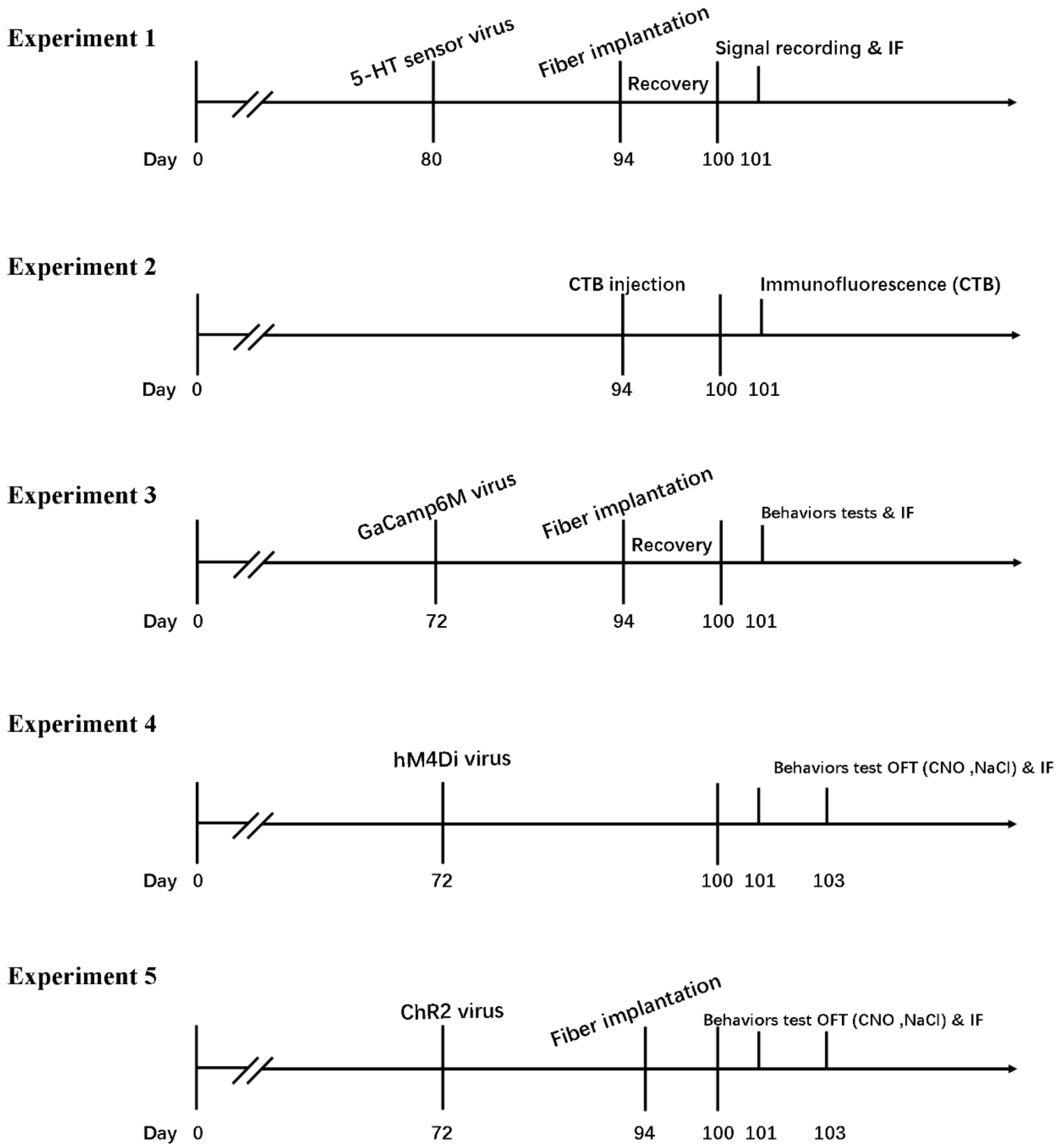

Figure S1 The scheme of the whole experiments.

IF: immunofluorescence; OFT: open field test.

Figure S2

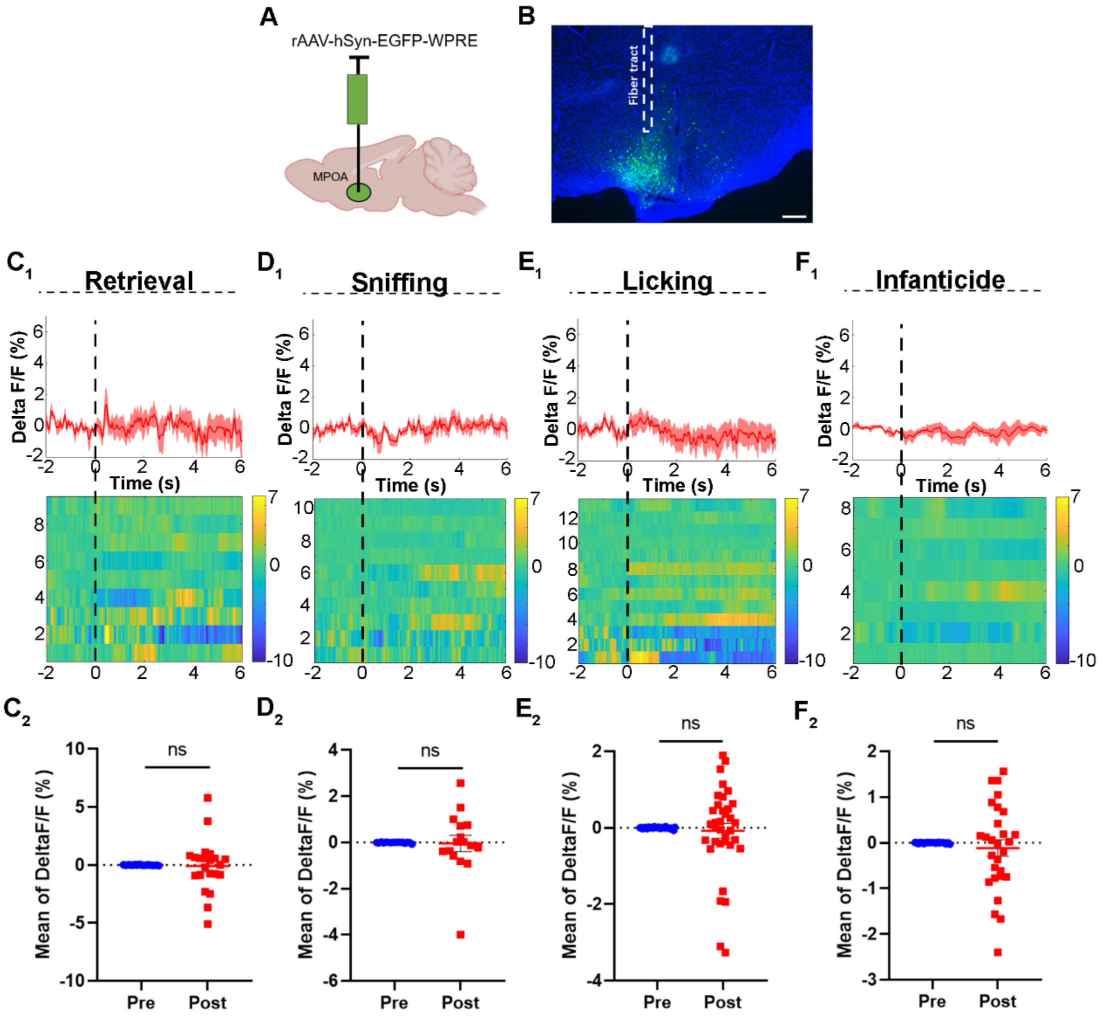

Figure S2 Change in fluorescence signal of control virus of 5-HT sensor 1.0 during different behaviors.

(A and B) The injection schematic and expression of eGFP virus. (C_1 – F_1) The results of retrieval, sniffing, licking, and infanticidal behavior of male voles. Up, the change of delta F/F accompany with the times. Down, the heat map of each behavior. C_1 n = 9; D1 n = 10; E_1 n = 13; F_1 n = 8. (C_2 – F_2) The results of the comparison of mean of delta F/F between pre and post of behavior. Paired sample t-test. C_2, n = 22, t (21) = 0.208, P = 0.837; D_2, n = 16, t (15) = 0.096, P = 0.925; E_2, n = 35, t (34) =0.407, P = 0.687; F_2, n = 28, t (27) = 0.625, P = 0.537. The data showed as mean ± SEM. Scale bar, 200 µm. ns, no significant.

Figure S3

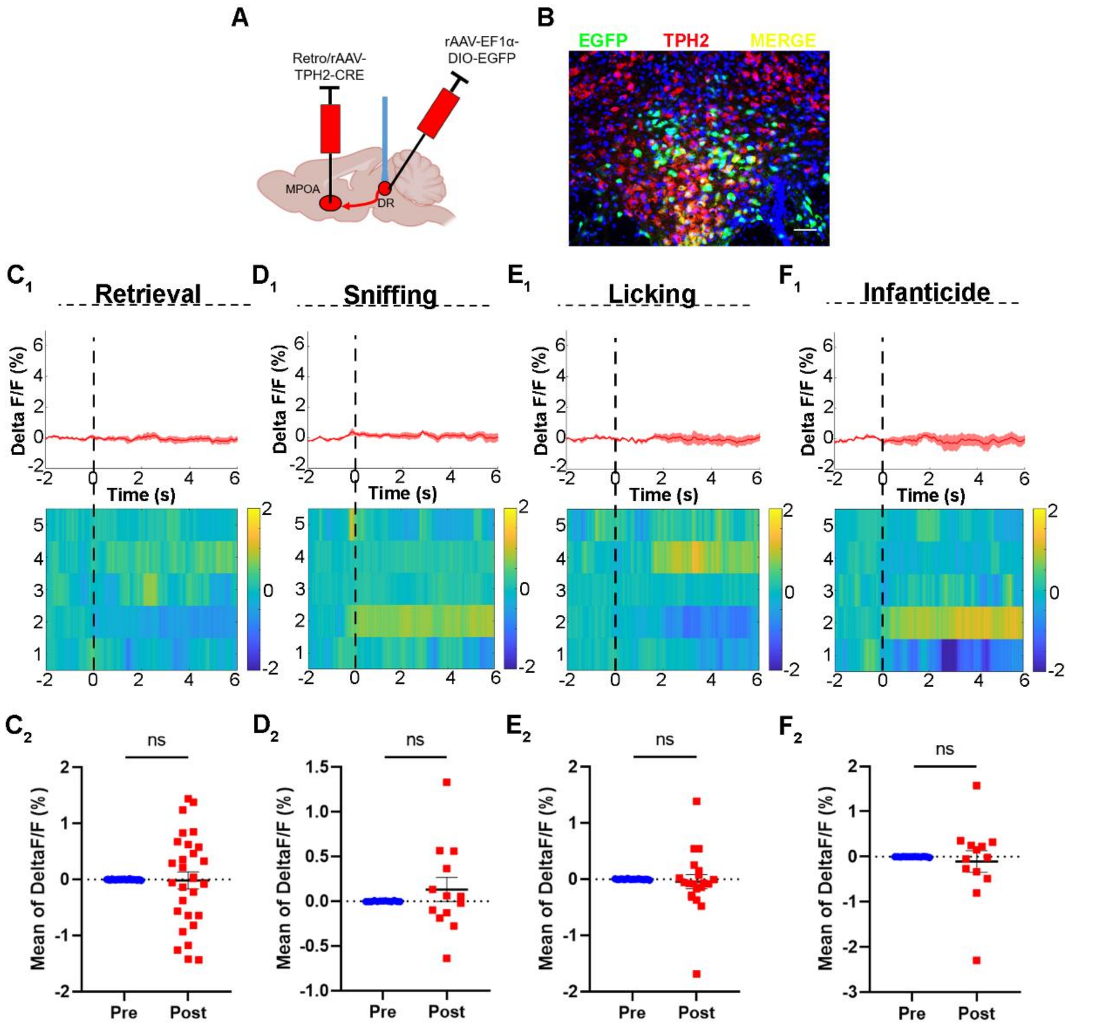

Figure S3 Change in fluorescence signal of the control virus of GCaMP6m during different behaviors.

(A and B) The injection strategy and expression of eGFP control virus. (C) Change in fluorescence signal upon retrieval behavior of paternal and infanticide voles. C1 represent delta F/F and heat map. n = 5 voles; C2, the mean of delta F/F, paired sample t-test, n = 29, t (28) = 0.090, P = 0.929 > 0.05. (D) Change in fluorescence signal upon sniffing behavior of paternal and infanticide voles. D_1 represent delta F/F and heat map. n = 5 voles; D_2, the mean of delta F/F, paired sample t-test, n = 13, t (12) = -0.974, P = 0.349 > 0.05. (E) Change in fluorescence signal upon of licking behavior of paternal voles. E_1 represent delta F/F and heat map. n = 5 voles; E_2, the mean of delta F/F, paired sample t-test, n = 20, t (19) = 0.326, P = 0.748 > 0.05. (F) Change in fluorescence signal upon killing pups of infanticide voles. F_1 represent delta F/F and heat map. n = 5 voles; F_2, the mean of delta F/F, paired sample t-test, n = 13, t (12) = 0.425, P = 0.679 > 0.05. The data showed as mean ± SEM. Scale bar, 200 μm. ns, no significant.

Figure S4

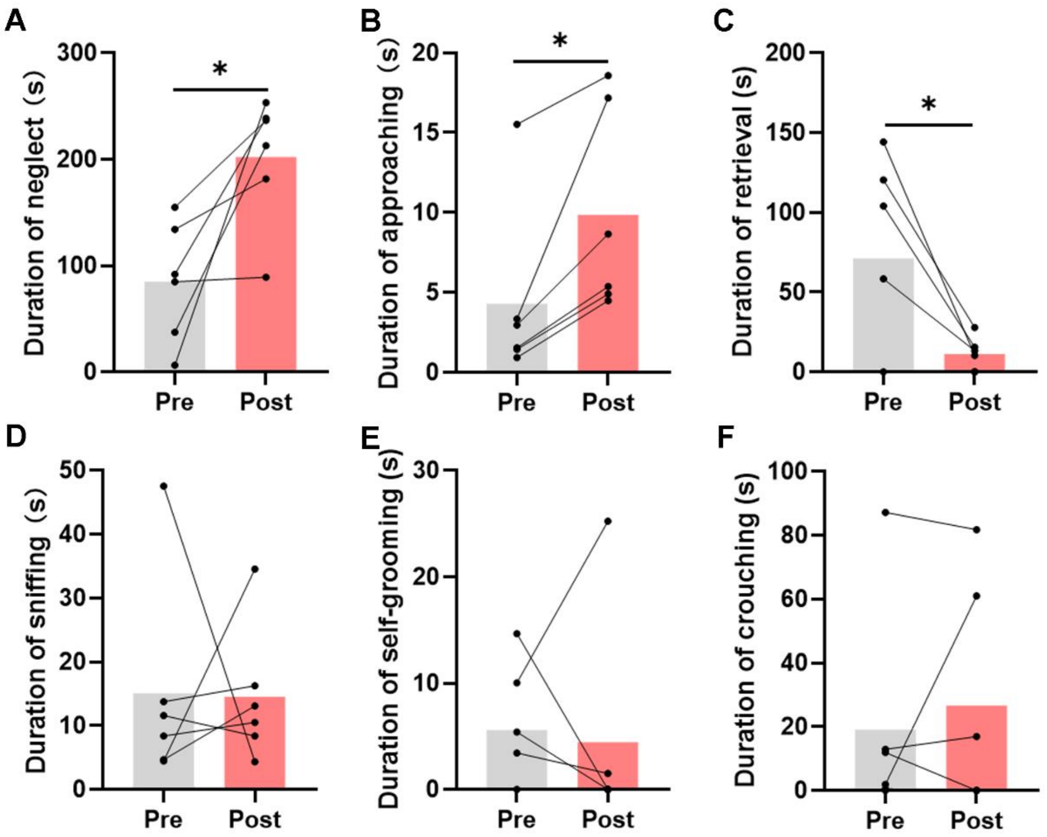

Figure S4 Effects of chemogenetic inhibition of DR to MPOA 5-HTergic projection on other behaviors.

(A) Effects of chemogenetic inhibition on neglecting of hM4Di-expressing voles before and after injecting CNO. Paired sample t-test. t (4) = -3.173, P = 0.034 < 0.05; (B) Effects of chemogenetic inhibition on approaching of hM4Di-expressing voles before and after injecting CNO. Paired sample t-test. t (4) = -2.964, P = 0.041 < 0.05; (C) Effects of chemogenetic inhibition on retrieval of hM4Di-expressing voles before and after injecting CNO. Paired sample t-test. t (4) = 3.15, P = 0.034 < 0.05; (D) Effects of chemogenetic inhibition on sniffing of hM4Di-expressing voles before and after injecting CNO t. Paired sample t-test. t (4) = 0.717, P = 0.513 > 0.05; (E) Effects of chemogenetic inhibition on self-grooming of hM4Di-expressing voles before and after injecting CNO. Paired sample t-test. t (4) = 1.599, P = 0.185 > 0.05; (F) Effects of chemogenetic inhibition on crouching of hM4Di-expressing voles before and after injecting CNO. Paired sample t-test. t (4) = -0.639, P = 0.558 > 0.05. The data showed as mean ± SEM. *, P < 0.05.

Figure S5

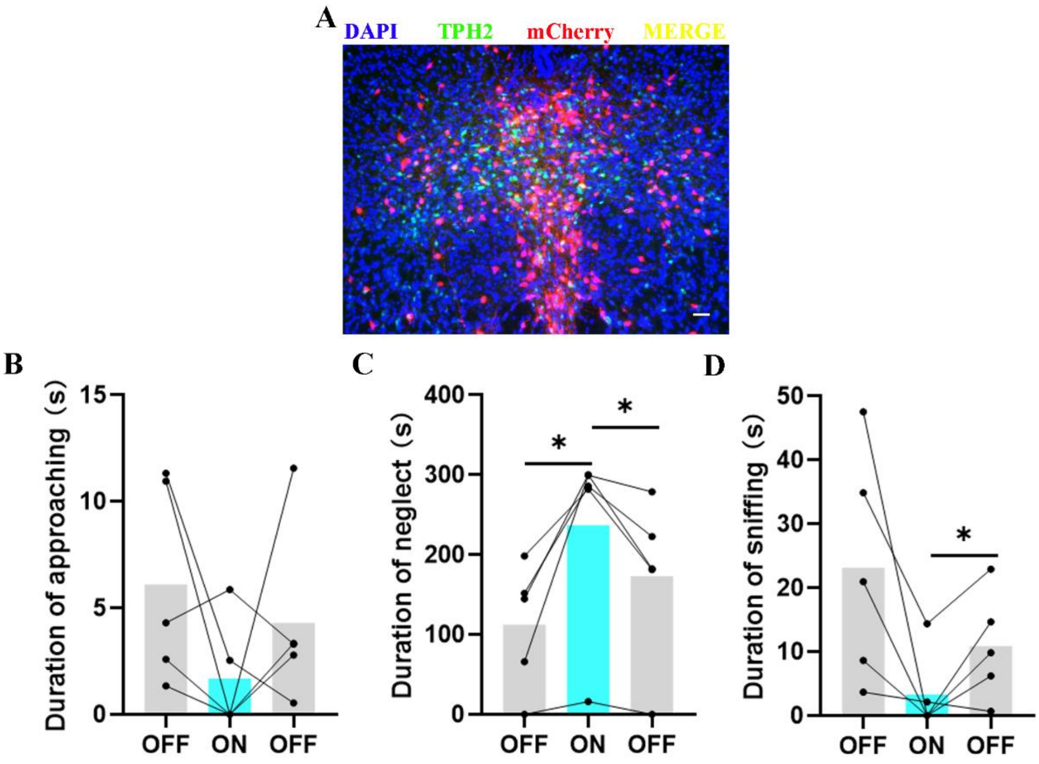

Figure S5 Effects of optogenetic activation of DR to MPOA 5-HTergic projection on other behaviors.

(A) The expression of mCherry virus in DR at the optogenetic activation experiment. (B) Effects of optogenetic activation on approaching behaviors of ChR2-experssion voles. Paired sample t-test. $t_{off\text{-}on}$ (4) = 1.883, P = 0.133 > 0.05; $t_{on\text{-}off}$ (4) =-1.036, P = 0.359 > 0.05. (C) Effects of optogenetic activation on neglecting behaviors of ChR2-expressing voles. Paired sample t-test. $t_{off\text{-}on}$ (4) = -3.429, P = 0.027 < 0.05; $t_{on\text{-}off}$ (4) =3.101, P = 0.036 < 0.05. (D) Effects of optogenetic activation on sniffing behaviors of ChR2-experssion voles. Paired sample t-test. $t_{off\text{-}on}$ (4) = 2.531, P = 0.065 > 0.05; $t_{on\text{-}off}$ (4) =-2.862, P = 0.046 < 0.05. The data showed as mean ± SEM. Scale bar, 200 μm. *, P < 0.05.

Figure S6

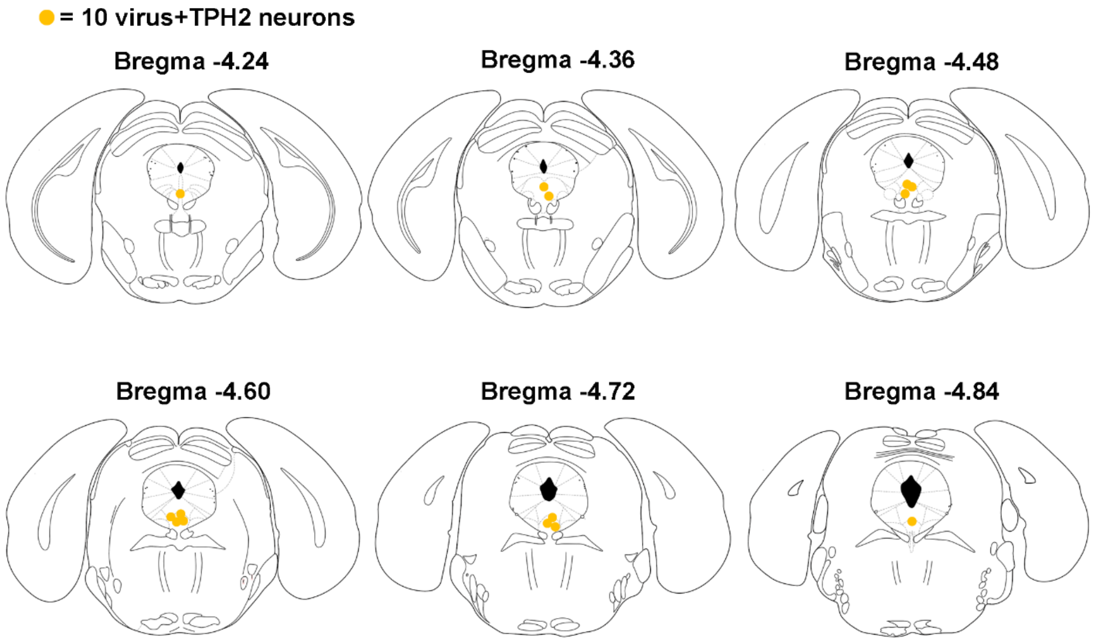

Figure S6 The injection sites and diffusion of virus in the ventral part of DR.
